# Synergistic Effects of *Beauveria bassiana* and Insecticides for Integrated Management of *Bactrocera dorsalis* (Hendel) (Diptera: Tephritidae)

**DOI:** 10.3390/insects16101067

**Published:** 2025-10-19

**Authors:** Xiaole Wang, Yunfei Li, Yuping Zha, Yubin Tian, Jing Wang, Hanbing Li, Zhihui Zhu, Wanlun Cai

**Affiliations:** 1 Hubei Insect Resources Utilization and Sustainable Pest Management Key Laboratory, College of Plant Science & Technology, Huazhong Agricultural University, Wuhan 430070, China; 17671797293@163.com (X.W.); alpha996lyf@163.com (Y.L.); t1428531645@126.com (Y.T.); wj1335699763@163.com (J.W.); 18331202139@163.com (H.L.); 2 Hubei Academy of Forestry, Wuhan 430070, China; zhayuping@163.com

**Keywords:** oriental fruit fly, entomopathogen, pesticides, integrated pest management, biocompatibility, synergy

## Abstract

*Bactrocera dorsalis* (Hendel) (Diptera: Tephritidae) is a significant agricultural pest that causes substantial economic losses globally. While chemical insecticides are commonly used for its management, increasing resistance and environmental concerns underscore the need for alternative control strategies. This study evaluated the potential of integrating the entomopathogenic fungus *Beauveria bassiana* strain Bb-33 (Hypocreales: Clavicipitaceae) with reduced doses of chemical insecticides for sustainable *B. dorsalis* management. The compatibility of *B. bassiana* Bb-33 with six commonly used insecticides—spinosad, emamectin benzoate, avermectin, thiamethoxam, beta-cypermethrin, and imidacloprid—was assessed. Among them, emamectin benzoate exhibited the least inhibitory effects on spore germination, mycelial growth, and sporulation of *B. bassiana*. Laboratory bioassays demonstrated synergistic interactions between *B. bassiana* Bb-33 and emamectin benzoate, particularly when the ratio of emamectin benzoate to *B. bassiana* exceeded 4:1, as indicated by co-toxicity coefficients greater than 100. However, greenhouse trials revealed that the combined formulation had lower efficacy in reducing *B. dorsalis* adult populations and pupal emergence rates compared to emamectin benzoate alone, though it was more effective than *B. bassiana* Bb-33 applied independently. Importantly, this composite formula reduces pesticide usage, which highlights its potential to mitigate environmental impacts. This study underscores the promise of integrating *B. bassiana* Bb-33 with reduced doses of emamectin benzoate as a viable strategy for managing *B. dorsalis*. Despite its current limitations in greenhouse trials, further optimization of formulation stability and application methods could enhance its field performance, offering an effective and environmentally friendly alternative to conventional chemical control methods.

## Simple Summary

*Bactrocera dorsalis* (Hendel) (Diptera: Tephritidae), commonly known as the oriental fruit fly, is a highly invasive and economically significant pest with a global impact. Current control strategies for *B. dorsalis* are largely dependent on chemical insecticide. However, the prolonged and widespread use of these chemicals has raised several concerns, such as resistance. In this study, integrating biological control agents with lower doses of chemical insecticides can address these limitations to some extent, but the compatibility of fungal agents with insecticides is a critical consideration. In this study we evaluated the biocompatibility of six commonly used insecticides with *B. bassiana* Bb-33, assessed the synergistic potential of compatible insecticide–fungus combinations against *B. dorsalis* under laboratory conditions, and investigated the efficacy of the most promising combination against *B. dorsalis* adults and pupae in greenhouse trials. We found that emamectin benzoate exhibited the lowest inhibition of *B. bassiana* spore germination and mycelial growth across different concentrations. Moreover, combination treatments of *B. bassiana* Bb-33 with emamectin benzoate demonstrated synergistic effects against *B. dorsalis* adults in laboratory bioassays. Greenhouse trials revealed that the combined treatment significantly reduced *B. dorsalis* adult populations and pupal emergence rates compared to individual treatments.

## 1. Introduction

*Bactrocera dorsalis* (Hendel) (Diptera: Tephritidae), commonly known as the oriental fruit fly, is a highly invasive and economically significant pest with global impact. Originally native to Asia, *B. dorsalis* has progressively expanded its distribution to encompass much of southern China and numerous other countries, leading to substantial economic losses in fruit and vegetable production worldwide [1,2]. A recent survey reported that the infestation rate of guava orchards in Guangzhou reached as high as 40% [3]. Its broad host range, high reproductive capacity, and strong adaptability to diverse climatic conditions have collectively consolidated its status as a major quarantine pest [4].

The damage caused by *B. dorsalis* is multifaceted. Female flies oviposit in fruits, causing mechanical damage that facilitates the invasion of secondary pathogens. Following hatching, larvae feed on fruit flesh, resulting in premature fruit drop and significant yield losses. Beyond direct crop losses, *B. dorsalis* infestation imposes broader economic consequences, including restricted market access and increased production costs associated with quarantine and control measures [5].

Current control strategies for *B. dorsalis* rely heavily on chemical insecticides, including direct spraying and the use of toxic baits to attract and kill adults [6]. However, prolonged and widespread reliance on insecticides has raised multiple concerns. Field populations of *B. dorsalis* have developed resistance to several major classes of insecticides [7]. Moreover, increasing attention has been drawn to the adverse environmental impacts and non-target effects of broad-spectrum chemicals [8]. These limitations underscore the urgent need to develop alternative, more sustainable control strategies. Compared with chemical insecticides, entomopathogenic fungi offer several advantages: they reduce environmental contamination, minimize negative impacts on beneficial organisms such as natural enemies, and pose a lower risk of resistance development.

Among biological control agents, entomopathogenic fungi, particularly *Beauveria bassiana* (Hypocreales: Clavicipitaceae), have attracted considerable interest as potential alternatives or complements to chemical control [9]. *B. bassiana* has been documented to infect over 700 insect species across various orders and can affect all life stages of *B. dorsalis* [10]. Nevertheless, the efficacy of *B. bassiana* under field conditions is variable due to environmental factors such as UV radiation, temperature, and humidity [11].

To address these limitations, researchers have proposed integrating biological control agents with lower doses of chemical insecticides. This approach, known as integrated pest management (IPM), aims to achieve effective pest control while minimizing environmental impacts and delaying resistance development [12]. Several studies have demonstrated synergistic effects when combining entomopathogenic fungi with sublethal doses of insecticides against various pests [13,14,15,16]. For instance, combining veratrol or azadirachtin with *B. bassiana* has shown significantly greater effectiveness against Aleyrodidae compared with single-agent treatments [15]. Despite such promising results, applications of insecticide–fungus combinations for controlling *B. dorsalis* remain relatively limited.

An important consideration in designing such integrated strategies is the compatibility between fungal agents and insecticides, as certain chemicals may inhibit fungal growth, sporulation, or infectivity [17]. Previous studies evaluating the effects of insecticides on *B. bassiana* have reported that lufenuron and ethoxyquin exert minimal inhibitory impacts, particularly when exposure times are short [18]. Selecting insecticides that are highly compatible with *B. bassiana* can further enhance the efficacy of combined treatments. Therefore, assessing the biocompatibility of potential insecticide partners is a critical prerequisite for developing effective IPM strategies.

Despite growing interest in this approach, research exploring the combined use of *B. bassiana* and insecticides specifically for controlling *B. dorsalis* remains limited. In this study, we aim to address this knowledge gap by (1) evaluating the compatibility of six commonly used insecticides with *B. bassiana* strain Bb-33; (2) assessing the synergistic potential of compatible insecticide–fungus combinations against *B. dorsalis* under laboratory conditions; and (3) investigating the efficacy of the most promising combination against adult and pupal stages in greenhouse trials. Our findings provide new insights into the identification of effective, compatible combinations of *B. bassiana* and insecticides, thereby contributing to the development of integrated and sustainable strategies for the management of *B. dorsalis*.

## 2. Materials and Methods

### 2.1. Test Strains and Pesticides

Entomopathogenic fungus *B. bassiana* Bb-33 was isolated from infected insects and obtained from Hubei Academy of Forestry Sciences. Detailed information regarding the pesticides used in this study is presented in Table 1 (The insecticides are pure active ingredients). The pesticides were obtained from preliminary laboratory screening and exhibit biocontrol activity against *B. dorsalis*. The *B. dorsalis* was provided by the Key Laboratory of Insects, Huazhong Agricultural University, and was reared under controlled conditions (26 ± 1 °C, 65 ± 5% RH, and a photoperiod of 14 h light:10 h dark, L:D = 14:10). Two generations were maintained for approximately 40 days before the experiments.

### 2.2. Determination of Median Lethal Concentration of Highly Virulent Strains

Spores were harvested using a 0.05% Tween-80 aqueous solution and prepared into a spore suspension at a concentration of 2 × 10^8^ conidia/mL. This suspension was then diluted to concentrations of 1 × 10^8^, 5 × 10^7^, 2 × 10^7^, and 2 × 10^6^ conidia/mL. Fifteen female and fifteen male adults were selected one day after eclosion and sprayed with 1 mL of spore suspension, while 0.05% Tween-80 aqueous solution was used as the control. The greenhouse conditions were maintained at a temperature of 26 ± 1 °C, relative humidity of 65 ± 5%, and a photoperiod of 14 h light and 10 h dark (L:D = 14:10). The mortality of adult insects was observed and recorded daily over a period of 10 days. The experiment was carried out in three independent sessions, with three replicates pear treatment in each experiment.

### 2.3. Assessment of Compatibility Between Beauveria bassiana and Insecticides

#### 2.3.1. Preparation of Insecticide Solutions

The original pesticides were dissolved in 10 mL of acetone solution. Subsequently, they were prepared according to the lethal concentration (LC_50_ (lethal)) with a 0.1% Triton solution, as specified in Table 1, and labeled as C. Further dilutions were made: fivefold (0.2 × LC_50_) and tenfold (0.1 × LC_50_). (Lethal concentration: The concentration of an agent that kills 50% of the test population required to cause the death of half of a population of organisms.) A 0.1% Triton supplemented with a suitable amount of acetone was prepared as the control (CK) treatments.

#### 2.3.2. Effect of Insecticides on Spore Germination of *B. bassiana* Bb-33

After culturing the Bb-33 on PDA medium for 12 days, spores were scraped off with an inoculation needle and added to a germination solution containing 0.05% Tween-80. Following centrifugation at 8000 rpm for 5 min at 24 °C, the spores were re-suspended in the germination solution using a magnetic stirrer. The concentration of the spore suspension was adjusted to 1 × 10^7^ conidia/mL. Different insecticides and the 0.1% Triton solution supplemented with acetone were added to the spore suspension at a 1:1 volume ratio, mixed thoroughly by shaking, and incubated on a shaking platform at 25 ± 1 °C (170 rpm) for 16 h. Three sets of replicates were performed for each treatment. Spore germination was observed under a microscope, and the germination inhibition rate was calculated. The experiment was repeated three times, with three technical replicates for each treatment. A control group, consisting of a spore suspension without insecticides, was included in the study.

Spore germination inhibition rate (%) = (1 − treatment group germination rate/control group germination rate) × 100%

#### 2.3.3. Effect of Insecticides on Colony Growth of *B. bassiana* Bb-33

To assess the colony growth inhibition rate of *B. bassiana* Bb-33, different insecticides were incorporated into the culture medium to create insecticide-enriched medium. The insecticides were tested at three concentration levels: LC_50_ (lethal), 0.2 × LC_50_ (sublethal), and 0.1 × LC_50_ (sub-sublethal). Insecticide solutions of different concentrations, along with the 0.1% Triton solution containing an appropriate amount of acetone prepared as described in Section 2.3.1, were filtered through a membrane, mixed with PDA medium at a volume ratio of 1:9, and then poured into culture dishes to ensure uniform distribution. A 10 μL aliquot of spore suspension (1 × 10^7^) was placed at the center of each plate, with three PDA plates prepared per treatment. A culture medium without insecticides served as the control. The plates were incubated at 25 ± 1 °C with a light cycle of L:D = 14:10. On the 10th day, the colony diameter for each treatment was measured using the cross method, and the growth inhibition rate was calculated. The experiment was repeated three times, with three technical replicates for each treatment.

Colony growth inhibition rate (%) = (1 − treatment group colony diameter/control group colony diameter) × 100%

#### 2.3.4. Effect of Insecticides on Spore Production of *B. bassiana* Bb-33

Colonies of *B. bassiana* Bb-33 cultivated on insecticide-enriched medium for 10 days (as described above) were used in this experiment. Samples were taken halfway between the center and the edge of the colony, approximately at half the diameter, and made into sections of fungal culture with a diameter of about 5 mm. Four sections of fungal culture were taken evenly from each toxic plate and placed in 20 mL of an aqueous solution containing 0.05% Tween-80. The sections of fungal culture were crushed using a glass rod, and the resulting spore suspension was filtered through a syringe containing absorbent cotton to remove mycelium and culture medium. The filtered spore suspension was mixed using a magnetic stirrer, and the spore concentration was measured with a hemocytometer. The experiment was repeated three times, with three technical replicates for each treatment.

Spore production inhibition rate (%) = (1 − treatment group spore yield/control group spore yield) × 100%

### 2.4. Synergistic Effect and Greenhouse Evaluation

#### 2.4.1. Toxicity of *B. bassiana* Combined with Chemical Insecticides Against *B. dorsalis*

Based on the LC_50_ values of individual insecticides against *B. dorsalis* (refer to Table 1), solutions with the LC_50_ (lethal) of insecticides and spore suspensions of *B. bassiana* Bb-33 were prepared. The insecticides and spore suspensions were mixed at volume ratios of 9:1, 4:1, 1:1, 1:4, and 1:9 to prepare stock solutions. Each of these stock solutions was subsequently diluted 10-fold, 5-fold, 3-fold, 2.5-fold, 2-fold, and 1.5-fold with sterilized distilled water to generate concentration gradients. The toxicity of the combined formulations against *B. dorsalis* was assessed in a laboratory setting using the spray method. One milliliter of the diluted solution was sprayed on the dorsal side of the selected *B. dorsalis* adults. Each group of consisted of 15 male and female adults, one day after emergence. The greenhouse conditions were maintained at 26 ± 1 °C, 65 ± 5% relative humidity, and a 14 h light: 10 h dark (L:D = 14:10). The mortality of adult insects was observed and recorded daily for 10 days. A control group was treated with a 0.05% Tween-80 aqueous solution, and calculated the LC_50_ of different ratios of the combined formulations. The experiment was conducted in three sessions, with three replicates of each treatment in each experiment.

The co-toxicity coefficient method [19] was employed to analyze the toxicity of the combined formulations against *B. dorsalis* and to calculate the co-toxicity coefficient (CTC). The toxicity of the combined formulations was evaluated based on the size of the coefficient. If the calculated CTC is less than 100, it indicates an antagonistic effect; if greater than 100, it implies a synergistic effect; and if approximately equal to 100, it signifies an additive effect.

Toxicity index = LC_50_ of standard agent/LC_50_ of tested agent × 100;

Actual toxicity index = LC_50_ of standard agent/LC_50_ of compound agent × 100;

Actual toxicity index = LC_50_ of standard agent/LC_50_ of compound agent × 100;

Theoretical toxicity index = Toxicity index of A × Content of A in the compound agent (%) + Toxicity index of B × Content of B in the compound agent (%);

Co-toxicity coefficient = Actual toxicity index/Theoretical toxicity index × 100

#### 2.4.2. Greenhouse Control Experiment of *B. dorsalis* Adults

The greenhouse control experiment was conducted in a glass greenhouse of Huazhong Agricultural University. Prior to the experiment, healthy potted *Citrus reticulata Blanco* plants were selected and placed inside insect-rearing nets. The distance between each pot of *citrus* is 50 cm. The greenhouse conditions were maintained at 26 ± 1 °C, 65 ± 5% relative humidity, and a 14 h light: 10 h dark photoperiod (L:D = 14:10). The *citrus* plants were divided into 4 treatments, the control, the *B. bassiana* Bb-33 spore liquid treatment, the emamectin benzoate single-agent treatment, and the emamectin benzoate and *B. bassiana* Bb-33 compound treatment. The respective treatments were treated by spraying with a plastic sprayer until the citrus leaves were moist (approximately 20 mL of liquid pesticide was sprayed on each pot of citrus). Each citrus plant was inoculated with 30 sexually mature *B. dorsalis* adults, comprising 15 females and 15 males. Three potted citrus plants were used for each treatment, and the experiment was independently repeated three times. The control (CK) was treated with a 0.05% Tween-80 solution and a 0.1% Triton mixed solution. From the second day to the tenth day post-inoculation, the number of adult *B. dorsalis* mortalities in each treatment area was recorded daily. The population reduction rate was calculated based on these data, and the number of fallen fruits was counted after the experiment.

Decline rate of population (%): (Number of living insects before treatment − Number of living insects after treatment)/Number of living insects before treatment × 100%;

Relative control effect (%): (1 − (Number of insects in the control group before treatment × Number of insects in the treatment group after treatment)/(Number of insects in the control group after treatment × Number of insects in the treatment group before treatment)) × 100%;

Fruit drop rate (%): Number of dropped fruits/Total number of fruits × 100%;

Infested fruit rate (%): Number of infested fruits/Total number of fruits × 100%

#### 2.4.3. Greenhouse Control Experiment of *B. dorsalis* Pupae

A layer of soil, 3–5 cm deep, was placed in storage boxes, and *B. dorsalis* pupae nearing eclosion were introduced into the soil. A 15 mL dose of the test solution was sprayed onto the soil surface, and the storage boxes were sealed with insect-proof netting and placed beside the citrus trees. The number of eclosed pupae was recorded. The treatments included *B. bassiana* Bb-33 spore liquid, emamectin benzoate single-agent, and emamectin benzoate and *B. bassiana* Bb-33 compound treatments. The control group (CK) received a 0.05% Tween-80 solution and a 0.1% Triton mixed solution. Each group contains 30 pupae with 3 replicates per group and three biological replicates.

Emergence rate (%): Number of emerging adults/Number of pupae × 100%

### 2.5. Data Analysis and Processing

Probit regression analysis was used to calculate the slope of the regression equation and the LC_50_ (median lethal concentration) of the reagent against *B. dorsalis*.

For the data from the experiment of assessing the effect of insecticides on Bb-33, data were analyzed using binomial generalized linear models (GLMs), and post hoc multiple comparisons were performed with Bonferroni correction. And the same analysis method was applied for the insect fruit rate, fruit falling rate, and emergence percentage data obtained from evaluation of a compound formulation for controlling *B. dorsalis* adults.

For the decline rate of population from evaluation of a compound formulation for controlling *B. dorsalis* adults, Kaplan–Meier survival curve with Log-rank test was applied and compared the difference between each two treatments. All the above statistical analyses were completed using the SPSS software package (version 22).

## 3. Results

### 3.1. Lethal Concentration of B. bassiana Bb-33

The LC_50_ of *B. bassiana* Bb-33 was determined to be 1.67 × 10^8^ conidia/mL, with a 95% confidence interval ranging from 1.21 × 10^8^ to 2.28 × 10^8^ conidia/mL. The regression slope was 1.72 ± 0.31, and the chi-square value was 4.81 (*df* = 2, *p* = 0.09), indicating that the model fit the observed mortality data well.

### 3.2. Effects of Pesticides on Spore Germination of B. bassiana Bb-33

Spore germination of *B. bassiana* Bb-33 was significantly influenced by pesticide type, pesticide concentration, and their interaction (in order: Wald χ^2^ = 124.26, *df* = 5, *p* < 0.001; Wald χ^2^ = 344.96, *df* = 2, *p* < 0.001, Wald χ^2^ = 204.88, *df* = 10, *p* < 0.001, respectively). As pesticide concentrations decreased, the inhibitory effects on spore germination also declined (Table 2). At the LC_50_ (sublethal) concentration, emamectin benzoate exhibited the lowest inhibition spore germination, whereas imidacloprid produced the highest inhibition. At the 0.2 × LC_50_ (sublethal) level, inhibition rates were generally around 10%; emamectin benzoate again displayed the lowest inhibition, while spinosad showed the highest. At the 0.1 × LC_50_ (sub-sublethal) concentration, inhibition rates for all tested pesticides were below 10%, and no significant differences were detected among treatments. Nevertheless, emamectin benzoate consistently exhibited the lowest inhibition, whereas thiamethoxam showed the highest. In summary, across all tested concentrations, emamectin benzoate consistently exerted the least inhibitory effect on the spore germination of *B. bassiana* Bb-33.

### 3.3. Colony Growth of B. bassiana Bb-33 Affected by Pesticides

The mycelial growth of *B. bassiana* Bb-33 was significantly influenced by pesticide type, pesticide concentration, and their interaction (in order: Wald χ^2^ = 21.79.26, *df* = 5, *p* = 0.001; Wald χ^2^ = 67.08, *df* = 2, *p* < 0.001, Wald χ^2^ = 18.67, *df* = 10, *p* = 0.048, respectively). The detailed inhibitory effects of different pesticides on *B. bassiana*’s mycelial growth are provided in Table 3. At the LC_50_ (lethal) concentration, the inhibition rates ranged from 10% to 20%, with thiamethoxam causing the highest inhibition, and beta-cypermethrin the lowest, followed closely by emamectin benzoate. At the 0.2 × LC_50_ (sublethal) concentration, emamectin benzoate exhibited the lowest inhibition rate, while imidacloprid showed the highest. At the 0.1 × LC_50_ (sub-sublethal) level, inhibition rates for all pesticides were below 10%. Notably, thiamethoxam, spinosad, and emamectin benzoate each showed inhibition rates below 5%. Overall, emamectin benzoate consistently demonstrated relatively low inhibitory effects on the mycelial growth of *B. bassiana* Bb-33 across all tested concentrations.

### 3.4. Sporulation Yield of B. bassiana Bb-33 Affected by Pesticides

Both pesticide type and concentration had significant effects on the sporulation yield of *B. bassiana* Bb-33, whereas their interaction was not significant (in order: Wald χ^2^ = 191.03, *df* = 5, *p* < 0.001, Wald χ^2^ = 261.55, *df* = 2, *p* < 0.001, Wald χ^2^ = 11.89, *df* = 5, *p* = 0.292, respectively). Detailed results are presented in Table 4. At the LC_50_ (lethal) concentration, emamectin benzoate exhibited the lowest inhibition of sporulation, significantly lower than all other pesticides tested. In contrast, imidacloprid and beta-cypermethrin produced the strongest inhibition. At the 0.2 × LC_50_ (sublethal) concentration, emamectin benzoate again exhibited the lowest inhibition rate, while beta-cypermethrin had the highest. At the 0.1 × LC_50_ (sub-sublethal) concentration, emamectin benzoate demonstrated the lowest inhibition rate, while imidacloprid and beta-cypermethrin displayed the highest rates. In summary, across all tested concentrations, emamectin benzoate consistently exerted the least inhibitory effect on the sporulation yield of *B. bassiana* Bb-33.

### 3.5. Screening of Pesticides with Good Compatibility with B. bassiana

The results indicated that spore germination of B. bassiana Bb-33 was minimally inhibited by emamectin benzoate, spinosad, and avermectin, with emamectin benzoate consistently exerting the weakest inhibitory effect. Specifically, inhibition rates for emamectin benzoate were 10.16% at LC_50_, 7.05% at 0.2 × LC_50_, and 5.18% at 0.1 × LC_50_. Although all tested pesticides influenced mycelial growth, emamectin benzoate consistently showed low inhibitory effects (12.26% at LC_50_, 6.39% at 0.2 × LC_50_, and 4.75% at 0.1 × LC_50_). Regarding sporulation, emamectin benzoate again exhibited the lowest inhibition rates, with reductions of 36.33% at LC_50_, 22.71% at 0.2 × LC_50_, and 12.24% at 0.1 × LC_50_. Taken together, these findings demonstrate that emamectin benzoate consistently exerted the least inhibitory effects on spore germination, mycelial growth, and sporulation of *B. bassiana* Bb-33, highlighting its favorable compatibility and suitability for integrated pest management (IPM) strategies.

### 3.6. Combined Toxicity and Co-Toxicity Coefficient of B. bassiana and Pesticides

The combined toxicity and co-toxicity coefficients of formulations containing *B. bassiana* Bb-33 and emamectin benzoate are summarized in Table 5. When the ratio of emamectin benzoate to *B. bassiana* exceeded 4:1, the co-toxicity coefficients of all formulations were greater than 100, indicating significant synergistic interactions. By contrast, ratios below 4:1 produced co-toxicity coefficients less than 100, reflecting antagonistic effects. To achieve maximum synergism while minimizing insecticide use, a 4:1 ratio of emamectin benzoate to *B. bassiana* Bb-33 was selected for subsequent greenhouse trials. Formulations at this ratio consistently yielded co-toxicity coefficients above 120, confirming its potential as an optimal combination for further evaluation.

### 3.7. Evaluation of a Compound Formulation for Controlling B. dorsalis Adults

**Figure 1:**
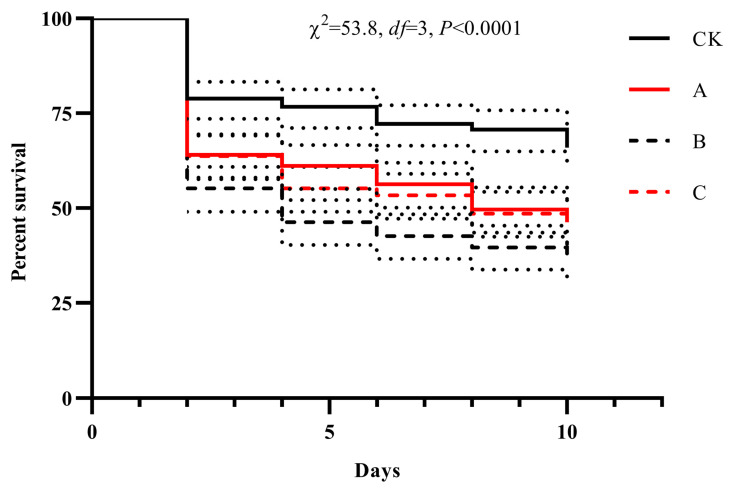
Survival dynamic of *Bactrocera dorsalis* adults treated by A (Bb33), B (Emamectin benzoate), C (Bb33 and Emamectin benzoate) and CK (0.05% TW-80 solution and 0.1% trition-100 mixture).

Based on the optimal ratio, a suspension containing 1.67 × 10^8^ conidia/mL of *B. bassiana* Bb-33 was combined with 1.77 mg/L of emamectin benzoate. Population reduction dynamics and overall control effects followed similar trends across all treatments, with efficacy increasing over time (Figure 1). Compared with the control (CK), all treatments had significant effects (Tables S1 and S2). However, the compound formulation (Bb-33 + emamectin benzoate) exhibited suboptimal performance relative to the individual treatments.

Significant differences were observed among treatments in both the percentage of citrus fruit infestation and the proportion of fruit drop (Wald χ^2^ = 81.63, *df* = 3, *p* < 0.001; Wald χ^2^ = 66.61, *df* = 3, *p* < 0.001, respectively). For both parameters, the compound formulation achieved only limited efficacy (Table 6). Notably, the overall control efficacy of all treatments including the compound formulation, emamectin benzoate alone, and *B. bassiana* spore suspension alone remained below 50%, indicating relatively poor control against adult *B. dorsalis*.

### 3.8. Evaluation of a Compound Formulation for Controlling B. dorsalis Pupae

Treatment formulations had significant effects on pupal emergence (Table 6; Wald χ^2^ = 23.61, *df* = 3, *p* < 0.001). The emergence rate of pupae treated with the compound formulation was 83.33%, significantly lower than that of the *B. bassiana* Bb-33 spore suspension alone but higher than that observed with emamectin benzoate treatment. Overall, the efficacy of all treatments against pupae was limited, likely due to the inherent resistance of *B. dorsalis* pupae and the restricted penetration of liquid applications. Nevertheless, delayed effects were observed: after emergence, a substantial proportion of adults from all treated groups died shortly thereafter. This suggests that while immediate control of pupae was insufficient, the treatments exerted delayed impacts on adult survival, thereby providing some degree of residual efficacy.

## 4. Discussion

*B. bassiana* is a widely used biological control agent that effectively suppresses harmful insects while posing minimal risk to humans, animals, and the environment. It is relatively easy to produce, store, and apply, offering substantial advantages for pest management programs. Previous studies have demonstrated that *B. bassiana* can significantly reduce populations of vector mosquitoes [20]. In contrast, chemical pesticides often exert detrimental effects on nontarget organisms, including natural enemies and other beneficial species, while also reducing microbial diversity [21]. Despite its advantages, the relatively slow infection process of *B. bassiana* and its high susceptibility to environmental factors frequently result in inconsistent control outcomes [22].

To address these limitations, compound formulations that integrate *B. bassiana* with low-toxicity insecticides, such as emamectin benzoate, have emerged as a promising strategy. Such combinations can accelerate pest mortality, enhance stability, reduce pesticide use, delay the development of resistance, and maintain environmental safety [23,24]. For instance, previous research demonstrated that the combined application of chlorantranili-prole and *Metarhizium anisopliae* achieved strong synergistic effects against *Spodoptera litura*, thereby reducing chemical input and mitigating resistance risks [25]. Nevertheless, compatibility between fungal pathogens and insecticides must be carefully assessed, as certain chemicals can negatively affect fungal growth and sporulation [26]. Reports indicate that some herbicides suppress *B. bassiana* germination and mycelial growth, ultimately reducing infectivity and dissemination. Interestingly, although herbicides altered the inducible immune response of *Tenebrio molitor*, they did not further affect survival under *B. bassiana* infection [27]. Similarly, insecticides have been shown to inhibit spore germination, slow hyphal growth, and decrease sporulation, with inhibitory effects often correlated with insecticide concentration [18,28].

In the present study, we demonstrated that emamectin benzoate exhibits high compatibility with *B. bassiana* Bb-33, causing only minimal inhibition of spore germination, mycelial growth, and sporulation. This compatibility is crucial for maintaining the effectiveness of compound formulations. Greenhouse assays further revealed that when the ratio of emamectin benzoate to *B. bassiana* Bb-33 exceeded 4:1, the co-toxicity coefficient surpassed 100, indicating synergism. Conversely, ratios of 1:1 or lower resulted in coefficients below 100, reflecting antagonistic interactions. Based on these results, a 4:1 ratio was selected as the optimal combination for further testing.

However, despite achieving a co-toxicity coefficient above 100 at this ratio, the compound formulation showed limited efficacy against *B. dorsalis* adults and pupae in greenhouse trials. This suboptimal performance may be partly attributable to the optimization method of Sun [19], which, although widely used, may be outdated and overly simplistic. Alternative approaches, such as Bliss independence (multiplicative mortality expectation), Loewe additivity, and response surface modeling, could provide more accurate predictions and more robust optimization for compound formulations in future studies. Furthermore, the poor efficacy observed against pupae may be linked to the limited penetration of liquid treatments and the intrinsic resistance of *B. dorsalis* at this developmental stage. Environmental fluctuations in humidity and temperature likely further reduced the viability and infectivity of *B. bassiana* spores. Interestingly, although immediate control efficacy was limited, delayed mortality was consistently observed in newly emerged adults, suggesting that compound formulations retain some residual effectiveness.

Numerous studies have demonstrated that compound formulations often exhibit notable synergistic effects compared to the single-agent treatments in practical field control [29]. Our findings partially align with this observation. Although the compound formulation of emamectin benzoate and *B. bassiana* Bb-33 demonstrated efficacy against *B. dorsalis* adults, its performance was slightly lower than that of emamectin benzoate alone. However, it was more effective than the single use of *B. bassiana*. In contrast to previous studies, this study is the first to conduct field-simulated experiments evaluating the control of *B. dorsalis* with compound formulations of insecticides and *B. bassiana*. This work provides a basis for the practical application of such compound formulations [30].

Despite these benefits, the lower control efficacy of the compound formulation compared to emamectin benzoate alone can be attributed to the environmental sensitivity of *B. bassiana*. Factors such as temperature, humidity, and UV exposure significantly affect spore germination, mycelial growth, and sporulation rates, ultimately impacting pest control outcomes [31]. This highlights a critical limitation of *B. bassiana*-based formulations, particularly under field conditions where environmental variables are less controllable. To address these challenges, the development of more stable formulations, such as wettable powders or oil-based suspensions, could enhance the viability and persistence of *B. bassiana* spores in adverse environmental conditions. Such advancements would protect spore activity and increase their stability, making *B. bassiana* a more robust option for integrated pest management [32].

Future research should focus on improving formulation stability through the selection of suitable carriers, wetting agents, and ultraviolet protectants, as well as refining application methods to achieve better coverage and penetration. Field trials will be essential to validate greenhouse findings and optimize application protocols under real-world conditions. For example, adjusting spray timing to align with *B. dorsalis* phenology could significantly improve control outcomes. Overall, while the combination of emamectin benzoate and *B. bassiana* Bb-33 shows promise as a strategy for reducing pesticide use and advancing environmentally sustainable pest management, further refinement is required to fully harness its potential.

## Figures and Tables

**Table 1:** Information of the Tested Chemical Pesticides.

Insecticide	Category	LC_50_ (mg/L)	Manufacturer
Spinosad 92%	Nicotinic Acetylcholine Receptor (nAChR) Allosteric Modulators	7.48	Huazhong Biotechnology Co., Ltd., Wuhan, China
Emamectin benzoate 95%	GABA Receptor Inhibitors	1.77	Huazhong Biotechnology Co., Ltd., Wuhan, China
Avermectin 96%	GABA Receptor Inhibitors	8.86	Huazhong Biotechnology Co., Ltd., Wuhan, China
Thiamethoxam 97%	Nicotinic Acetylcholine Receptor (nAChR) Agonists	71.09	Huazhong Biotechnology Co., Ltd., Wuhan, China
Beta-cypermethrin 96%	Voltage-Gated Sodium Channel Inhibitors	112.95	Huazhong Biotechnology Co., Ltd., Wuhan, China
Imidacloprid 96%	Nicotinic Acetylcholine Receptor (nAChR) Agonists	255.99	Huazhong Biotechnology Co., Ltd., Wuhan, China

**Table 2:** Effects of Chemical Insecticides on Spore Germination of *B. bassiana* Bb-33.

Insecticide	Spore Germination Inhibition Rate (%) ^a^		
	LC_50_ (Lethal)	0.2 × LC_50_ (Sublethal)	0.1 × LC_50_ (Sub-Sublethal)
Emamectin benzoate	10.16 ± 0.64 D	7.05 ± 0.48 A	5.18 ± 0.70 A
Spinosad	13.15 ± 0.38 D	11.11 ± 0.21 A	7.13 ± 0.61 A
Beta-cypermethrin	46.71 ± 1.17 B	9.03 ± 0.84 A	7.31 ± 0.33 A
Avermectin	15.00 ± 0.75 D	7.54 ± 1.00 A	7.30 ± 1.08 A
Imidacloprid	97.39± 0.61 A	10.20 ± 0.23 A	5.43 ± 0.74 A
Thiamethoxam	26.89± 1.02 C	8.15 ± 0.51 A	7.46 ± 0.22 A

^a^ The data in the table represent the mean value ± standard error. Mean values in a column followed by different uppercase case letters are significantly different on the basis of binomial GLMs with Bonferroni correction (α = 0.05).

**Table 3:** Effects of Chemical Insecticides on Mycelial Growth of *B. bassiana* Bb-33.

Insecticide	Mycelial Growth Inhibition Rate (%) ^a^		
	LC_50_ (Lethal)	0.2 × LC_50_ (Sublethal)	0.1 × LC_50_ (Sub-Sublethal)
Emamectin benzoate	12.26 ± 0.81 A	6.39 ± 0.27 B	4.75 ± 0.51 A
Spinosad	13.00 ± 1.20 A	9.51 ± 1.55 AB	3.45 ± 0.77 A
Beta-cypermethrin	11.71 ± 0.94 A	12.01 ± 0.58 AB	8.18 ± 0.53 A
Avermectin	14.75 ± 0.66 A	10.96 ± 0.98 AB	7.84 ± 0.65 A
Imidacloprid	15.67 ± 0.64 A	13.31 ± 0.61 A	8.18 ± 1.42 A
Thiamethoxam	16.95 ± 1.13 A	7.96 ± 1.69 AB	3.10 ± 1.07 A

^a^ The data in the table represent the mean value ± standard error. Mean values in a column followed by different uppercase case letters are significantly different on the basis of binomial GLMs with Bonferroni correction (α = 0.05).

**Table 4:** Effects of Chemical Insecticides on Sporulation of *B. bassiana* Bb-33.

Insecticide	Sporulation Inhibition Rate (%) ^a^		
	LC_50_ (Lethal)	0.2 × LC_50_ (Sublethal)	0.1 × LC_50_ (Sub-Sublethal)
Emamectin benzoate	36.33 ± 1.19 C	22.71 ± 0.65 D	12.24 ± 1.21 D
Spinosad	47.88 ± 0.75 BC	38.02 ± 3.39 B	27.84 ± 1.86 BC
Beta-cypermethrin	63.19 ± 1.89 A	46.95 ± 1.08 AB	34.37 ± 2.46 AB
Avermectin	54.11 ± 1.20 AB	39.10 ± 1.09 BC	24.54 ± 1.51 BC
Imidacloprid	63.63 ± 0.31 A	53.68 ± 1.82 A	39.50 ± 1.90 A
Thiamethoxam	52.97 ± 1.36 A	29.77 ± 1.10 CD	22.24 ± 2.43 C

^a^ The data in the table represent the mean value ± standard error. Mean values in a column followed by different uppercase case letters are significantly different on the basis of binomial GLMs with Bonferroni correction (α = 0.05).

**Table 5:** Combined Toxicity and Co-Toxicity Coefficient of Compound Agent on *B. dorsalis*.

Ratio	Slope ± SE	LC50 (mg/L)	95% Confidence Interval	Chi-Square Test	CTC
A ^a^:B ^b^ = 1:9	8.16 ± 1.03	1.11	0.99–1.21	2.48	175.11
A:B = 1:4	12.77 ± 1.75	1.74	1.59–1.86	1.26	123.87
A:B = 1:1	7.95 ± 0.69	5.33	4.91–5.75	0.45	60.05
A:B = 4:1	3.76 ± 3.00	9.40	6.15–11.53	1.69	66.12
A:B = 9:1	1.43 ± 1.00	18.70	——	0.93	48.44

^a^ *B. bassiana* Bb-33. ^b^ Emamectin benzoate.

**Table 6:** Fruit Injury and the Emergence of *B. dorsalis* Pupae in Different Treatment Areas.

Treatment Area	Insect Fruit Percentage ^e^	Fruit Falling Percentage ^e^	Emergence Percentage ^e^
A ^a^	18.47 ± 12.47 B	62.35 ± 0.12 B	91.11 ± 0.64 A
B ^b^	9.80 ± 1.56 C	51.08 ± 0.14 C	79.26 ± 0.98 B
C ^c^	16.05 ± 8.07 B	57.65 ± 0.03 BC	83.33 ± 0.64 B
CK ^d^	36.00 ± 5.72 A	79.72 ± 0.05 A	91.48 ± 0.74 A

^a^ Bb-33 suspension treatment. ^b^ Emamectin benzoate treatment. ^c^ Emamectin benzoate and Bb-33 compound agent treatment. ^d^ 0.05% TW-80 solution and 0.1% trition-100 mixture treatment. ^e^ The data in the table is the average value ± standard error. Mean values in a column followed by different uppercase case letters are significantly different on the basis of binomial GLMs with Bonferroni correction (α = 0.05).

## Data Availability

All relevant summarized data are included within the article. Raw datasets supporting the findings of this study are available from the corresponding author upon reasonable request.

## Supplementary Materials

The following supporting information can be downloaded at: https://www.mdpi.com/article/10.3390/insects16101067/s1, Table S1: Comparison of significance of survival curves for different treatments; Table S2: Sample sizes at risk.
