# Supplementary material for: Synergistic Effects of Beauveria bassiana and Insecticides for Integrated Management of Bactrocera dorsalis (Hendel) (Diptera: Tephritidae)
Source: Insects. 2025 Oct 19;16(10):1067. doi: 10.3390/insects16101067 (PMC12564208; doi:10.3390/insects16101067)
Supplement: Supplementary file 1 [file insects-16-01067-s001.zip › insects-3795239-supplementary.pdf]

Supplymentary Table S1 Comparison of significance of survival curves for different treatments

	A	B	C	CK
A		7.38	0.12	21.80
B	0.0066		5.54	53.49
C	0.7305	0.0186		24.87
CK	<0.0001	<0.0001	<0.0001	

Note: A, Bb-33 suspension treatment. B, Emamectin benzoate treatment. C, Emamectin benzoate and Bb-33 compound agent treatment. CK, 0.05% TW-80 solution and 0.1% trition-100 mixture treatment. The triangular data on the table represents the chi square values of the comparison between the horizontal and vertical treatment with Log rank test (α=0.05), while the lower triangular data represents the corresponding significance values.

Supplementary table S2 Sample sizes at risk

	Total observaitons	2d	4d	6d	8d	10d
CK	270	213	207	195	191	178
A	270	173	165	152	134	125
B	270	149	125	115	107	96
C	270	172	149	144	131	122

Note: A, Bb-33 suspension treatment. B, Emamectin benzoate treatment. C, Emamectin benzoate and Bb-33 compound agent treatment. CK, 0.05% TW-80 solution and 0.1% trition-100 mixture treatment.
